# An Areal Isotropic Spline Filter for Surface Metrology

**DOI:** 10.6028/jres.120.006

**Published:** 2015-04-01

**Authors:** Hao Zhang, Mingsi Tong, Wei Chu

**Affiliations:** 1 College of Mechanical and Electronic Engineering, Nanjing Forestry University, Nanjing, 210037, China; 2 National Institute of Standards and Technology, Gaithersburg, MD 20899; 3 School of Mechatronics Engineering, Harbin Institute of Technology, Harbin, 150001, China

**Keywords:** areal filter, Gaussian filter, high-order spline filter, isotropic characteristic

## Abstract

This paper deals with the application of the spline filter as an areal filter for surface metrology. A profile (2D) filter is often applied in orthogonal directions to yield an areal filter for a three-dimensional (3D) measurement. Unlike the Gaussian filter, the spline filter presents an anisotropic characteristic when used as an areal filter. This disadvantage hampers the wide application of spline filters for evaluation and analysis of areal surface topography. An approximation method is proposed in this paper to overcome the problem. In this method, a profile high-order spline filter serial is constructed to approximate the filtering characteristic of the Gaussian filter. Then an areal filter with isotropic characteristic is composed by implementing the profile spline filter in the orthogonal directions. It is demonstrated that the constructed areal filter has two important features for surface metrology: an isotropic amplitude characteristic and no end effects. Some examples of applying this method on simulated and practical surfaces are analyzed.

## 1. Introduction

For a long time, measurement and evaluation of surface topography was mainly studied in two-dimensions (2D) due to the limits of measurement systems and instruments, signal processing techniques, and calculation capabilities of microprocessors. In 2D surface measurement, data are acquired and analyzed along a profile. Profiles contain less information than areal images and indicate in only a limited way the comprehensive characteristics and functional performance of surfaces. As described in Refs. [1,2], surface metrology is currently undergoing a major paradigm shift. The applications for 3D measurement of surfaces are becoming more and more common [3]. Certainly, the measurement and evaluation carried out from a 3D perspective can enhance both the analysis of surface topography and the control of surface manufacturing.

Filtration is a critical process used to separate surface roughness from finer fluctuations and from the waviness or to separate waviness from roughness [4]. As an important part of surface metrology, the research of filtration and its focus are undergoing a shift from 2D methods to 3D methods due to the increasing demand for areal characterization in both academia and industry. Several research groups have been working in this direction to promote the development of areal filtering technology [2,3,5,6]. Furthermore, the International Organization for Standardization (ISO) Technical Committee TC 213 is working on areal filtering standards as parts 60, 61, 62, and 69 in ISO/TS 16610.

Currently, the most commonly used filtering technique for 3D data is still the classical Gaussian filter because of its isotropic and phase correct transmission characteristic [2,5,7,8]. It is well known that the Gaussian filter always causes serious end effects [9], to the extent that the profile ends must be excluded subsequent to the filtering process. In order to overcome this problem, the ISO/TS 16610-22 standard recommended the spline filter as one substitute for the Gaussian filter [10,11]. Although the spline filter possesses such advantages as end preserving, fast calculation speed and good form following, it has not been accepted widely in the measurement of 3D surface because the application of the standard spline filter [10] to 3D data in orthogonal directions is demonstrated to be severely anisotropic [3].

This paper proposes a simple areal filtering algorithm based on the high-order spline filter. The algorithm can be implemented through successive application of a profile Gaussian filter in orthogonal directions. It possesses both desired features of isotropic amplitude characteristic and no end effects, and therefore is a feasible solution for areal filtering. Some application examples are given to verify the practicability of the new filter.

## 2. High-Order Spline Filter

According to Refs. [12,13], the classical variational method provides a penalty function to be minimized that is made up of two parts. One part is the *L*_2_ norm of the residual error guaranteeing the result’s closeness to the profile *z* [14]. The other part, called the bending energy, helps to ensure appropriate smoothness of the filtered result. A regularization parameter is used to control the compromise between closeness to the data and amount of smoothness. In Ref. [11], a spline filtering algorithm based on the variational principle is developed as an approach for the Gaussian filter. A first-order derivative term is added into the bending energy part to improve the transmission characteristic of the filter as a function of spatial frequency. With adjustment of the regularization parameters, this approach provides a good solution close to the filtering characteristic of the Gaussian filter. For this reason, the solution is called *an approximating filter*. Inspired by this idea, if more derivative items are added into the variational function, a high-order spline filter can be constructed to a better approximation [15].

$$\varepsilon =\sum_{i=1}^{N}{\left(z_{i}-w\left(x_{i}\right)\right)}^{2}+\int_{x_{1}}^{x_{N}}\left[\mu_{1}{\left(\frac{dw\left(x\right)}{dx}\right)}^{2}+\cdots +\mu_{n}{\left(\frac{d^{n}w\left(x\right)}{dx^{n}}\right)}^{2}\right]dx\to \text{Min}$$ (1)

where *z_i_* are the measured profile data with a constant sampling interval Δ*x*, *w*(*x*) is the output profile, *i* is the index of a point in the dataset, *N* is the total number of measured data points, and *μ_l_* (*l* = 1, ⋯, *n*) are the regularization parameters, *n* + 1 is defined as the order of the filter. From the above equation, it can be visualized that the standard spline filter [10] is a simplified version of Eq. (1) by removing the high-order derivative terms in the bending energy part.

Generally, the solution to the variational function of Eq. (1) can be solved by the matrix factorization algorithm [16], and the essential matrix equation is written as

(I+Q)W=Z (2)

where ***I*** is the identity matrix, ***Z*** is the vector of sampled data values, and ***W*** is the vector of output data values. Actually, the matrix equation is derived by performing the partial derivative operation of Eq. (1) with respect to *w*(*x_i_*). The coefficient matrix ***Q*** varies with different boundary conditions, which may be classified as periodic or non-periodic [10].

For the case of a periodic boundary condition, each row of Eq. (2) represents a solution equation with respect to *w_i_*,

$$\frac{\partial \varepsilon }{\partial w_{i}}=-2\left(z_{i}-w_{i}\right)+\left[-2\mu_{1}\left(\nabla^{2}w_{i}\right)+2\mu_{2}\left(\nabla^{4}w_{i}\right)+\cdots +{\left(-1\right)}^{n}2\mu_{n}\left(\nabla^{2n}w_{i}\right)\right]=0$$ (3)

where *w_i_* is the abbreviation of *w*(*x_i_*), and ∇ is the differential operator used to obtain finite differential approximations to each order derivative of *w_i_* and to simplify the whole differential equation.

$$\nabla^{m}s_{i}=\sum_{k=0}^{m}\left({}_{k}^{m}\right){\left(-1\right)}^{k}\cdot s_{i+\left\lfloor m/2\right\rfloor -k}$$ (4)

where $\left({}_{k}^{m}\right)$ are binomial coefficients.

The detailed analysis of the spline filter is provided by Goto et al. [14] and Johannes et al. [17]. Here, Eq. (3) is used to deduce the transfer function of the spline filter by the aid of a *z* transform, which includes a series of the second-order differential operator ∇^2^*^l^*, *l* = 1,* ⋯, n.* The *z* transform of ∇^2^*^l^* can be expressed as (z − 2 + z^−1^)*^l^*. Hence, the transfer function of the spline filter can be written as

$$G\left(z\right)=\frac{1}{1-\mu_{1}\left(z-2+z^{-1}\right)+\mu_{2}{\left(z-2+z^{-1}\right)}^{2}+\cdots +{\left(-1\right)}^{n}\mu_{n}{\left(z-2+z^{-1}\right)}^{n}}.$$ (5)

Furthermore, replacing the factor z by exp (−*jω*) and utilizing Euler’s formula, we arrive at

$$G\left(\omega \right)=\frac{1}{1+2\mu_{1}\left(1-\cos\omega \right)+4\mu_{2}{\left(1-\cos\omega \right)}^{2}+\cdots +2^{n}\mu_{n}{\left(1-\cos\omega \right)}^{n}}.$$ (6)

**Fig. 1:**
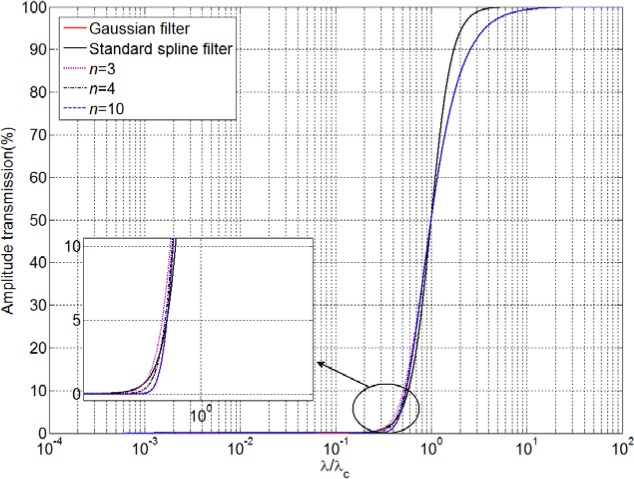
Transmission characteristics of the Gaussian filter, standard spline, and three high-order spline filters. (In the graph, the curves for *n* = 3, 4, and 10 and the Gaussian filter are indistinguishable in most parts.)

Obviously, Eq. (6) formulates the transmission characteristics of the high-order spline filters with arbitrary order. Here, *μ_l_* can be determined through a standard Taylor series expansion [15], which ensures that the transmission characteristic of a high-order spline filter can approximate that of the Gaussian filter with high accuracy. Figure 1 shows the transmission characteristics of several spline filters [15]. It illustrates that the greater the order numbers of the spline filter, the closer the approximation to the Gaussian filter.

## 3. The Areal Spline Filters

As mentioned in Refs. [1,3,18,19], the classical Gaussian filter has many excellent properties, such as zero-phase characteristic, minimum product of time width and frequency width, isotropic characteristic, and perfect separability of the 3D Gaussian function into filters operating in the *x*- and *y*-directions. The separability may contribute to simplifying the theoretical derivation of the each filtering algorithm and to speeding up the calculation. Moreover, the separability feature also gives us the inspiration for an indirect approach to design and implement a 3D spline filter. Based on this idea, we develop a novel areal spline filter with an isotropic amplitude characteristic, starting from the high-order spline filter which approximates to the Gaussian filter.

### 3.1 Separability of the Areal Gaussian Filter

The amplitude transfer function of the areal Gaussian filter is given by [20]

$$H\left(\lambda_{x},\lambda_{y}\right)=\exp\left\{-\pi \alpha \left[{\left(\frac{\lambda_{xc}}{\lambda_{x}}\right)}^{2}+{\left(\frac{\lambda_{yc}}{\lambda_{y}}\right)}^{2}\right]\right\}$$ (7)

where *α* = ln2/π and *λ_xc_*, *λ_yc_* are the cut-off wavelengths in the *x*- and *y*-directions respectively. The filter has an attenuation ratio of 50 % at the cut-off wavelength, for example, at *λ_x_* = *λ_xc_* and *λ_y_* = ∞ or at *λ_y_* = *λ_yc_* and *λ_x_* = ∞.

**Fig. 2:**
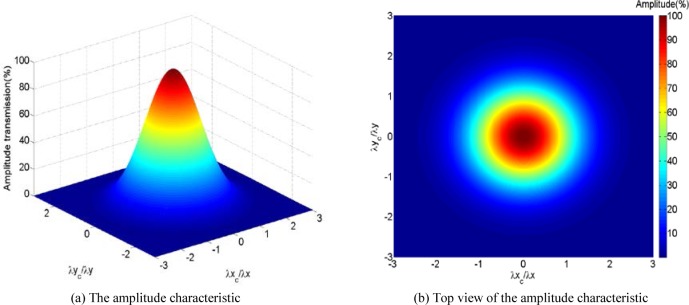
The amplitude characteristic of the areal Gaussian filter.

The separability of the areal Gaussian function can be described as

$$H\left(\lambda_{x},\lambda_{y}\right)=\exp\left[-\pi \alpha {\left(\frac{\lambda_{xc}}{\lambda_{x}}\right)}^{2}\right]\cdot \exp\left[-\pi \alpha {\left(\frac{\lambda_{yc}}{\lambda_{y}}\right)}^{2}\right]=H\left(\lambda_{x}\right)\cdot H\left(\lambda_{y}\right)$$ (8)

where *H*(*λ_x_*) and *H*(*λ_y_*) are exactly the same as the profile Gaussian function. Therefore, an areal Gaussian filter is equivalent to a profile Gaussian filter in the *x* direction followed by the profile Gaussian filter in the *y* direction. Figure 2 shows the amplitude characteristic of Eq. (7), which depicts the performance of rotational invariance. The areal Gaussian filter is clearly isotropic if the constants *λ_xc_* and *λ_yc_* are equal.

### 3.2 Design of the Areal Spline Filter

**Fig. 3:**
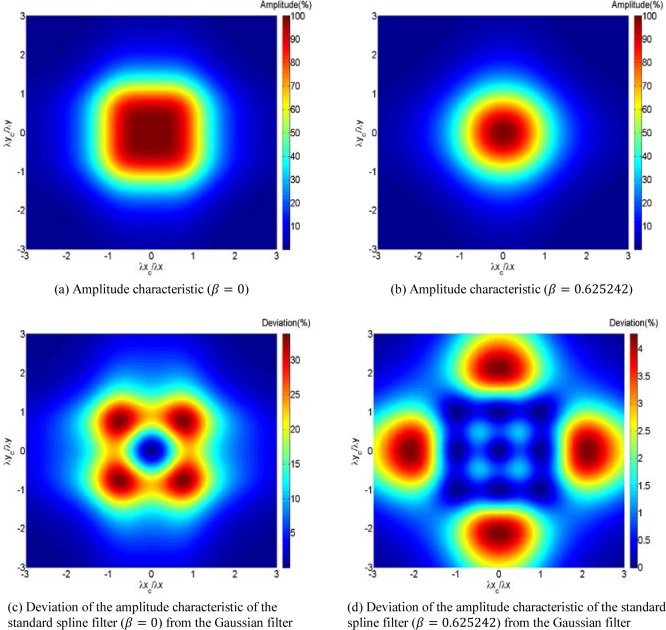
The amplitude characteristics of the areal spline filters.

The standard spline filter [10] can be extended directly for 3D data in the orthogonal directions. The amplitude characteristics of the areal spline filter are shown in Fig. 3, where Fig. 3 (a) is *β* = 0 and (b) is *β* = 0.625242. *β* called the tension parameter controls how tightly the spline curve fits through the data points [10]. The tension parameter also objectively controls how closely the transmission characteristic of the profile spline filter approximates that of the Gaussian filter. When *β* = 0.625242, the best approximation to a Gaussian filter is achieved. From Figs. 3 (c) and (d), it can be found that the closer the transmission characteristic between the profile spline filter and the Gaussian filter, the stronger the isotropic characteristic of the corresponding areal spline filter.

**Fig. 4:**
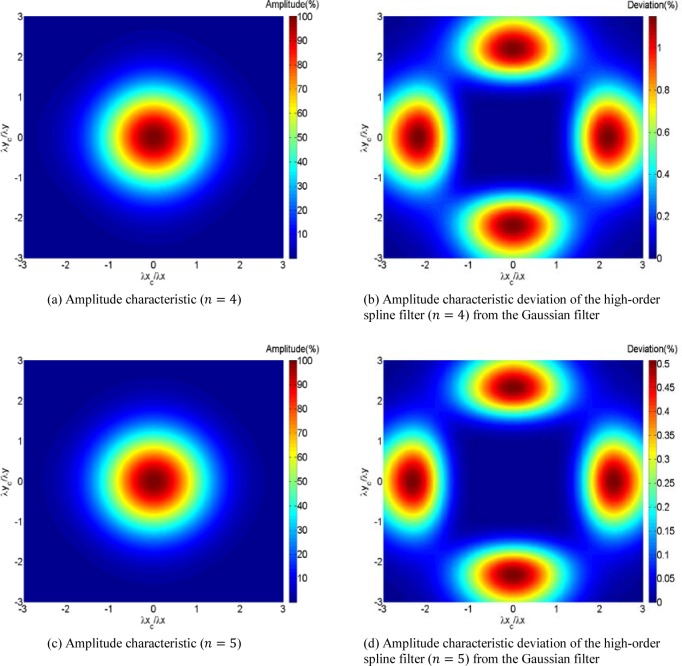
The amplitude characteristics of the high-order spline filters.

From the analysis above, it is feasible to approximate an isotropic amplitude characteristic of the areal spline filter by implementing an approximating spline filter in a manner similar to the separable areal Gaussian filter. Thus, based on a high-order spline filter, we construct an areal spline filter which provides a close approximation to an isotropic transmission characteristic. As expected, the higher the order, the better the isotropy of the transmission characteristic of the corresponding areal filter. Figures 4 (a) and (c) show the amplitude characteristics of the high-order spline filter of *n* = 4 and *n* = 5, and Figs. 4 (b) and (d) show their deviations from the Gaussian filter. These figures illustrate that the maximum bias between the high-order spline filter *n* = 4 and the Gaussian filter is 1.1493 %, while the maximum bias for high-order spline filter *n* = 5 is 0.5069 %. Although higher order spline filters can be created to achieve an even closer transmission characteristic to that of the Gaussian filter, in practice, excessive higher orders would result in slower computing speed. Hence, to balance between efficiency and effectiveness, we show examples for the filter with order 5 (that is, *n* = 4), whose deviation is completely acceptable for most requirements.

Beside the isotropic characteristic, the high-order spline filter also inherits the feature of no end effects owing to the application of the complete matrix procedure instead of the convolution between data and discrete filter. It does not need extra data at the profile ends during the process and hence does not cause significant end error. That merit is the principal reason why we promote the application of the areal spline filter in surface metrology.

## 4. Experiments and Comparison

### 4.1 Computer Simulation

**Fig. 5:**
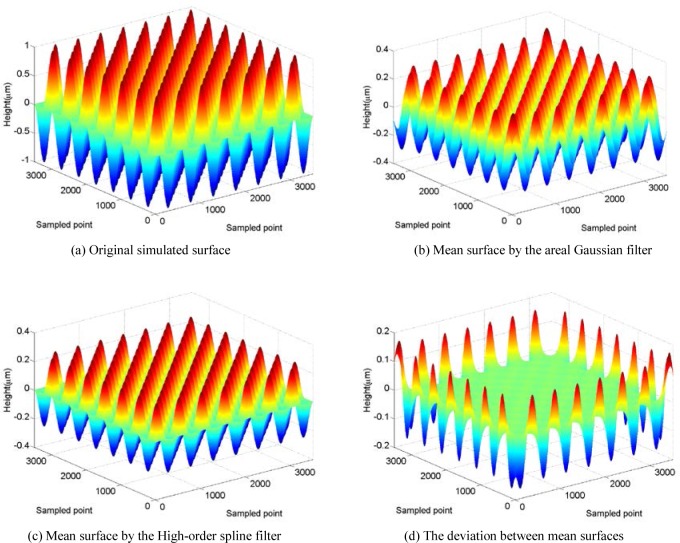
Simulated fundamental wavelength surface.

Comparing filtering results for a standard surface is helpful to highlight the advantages or shortcomings of an areal filtering algorithm. A simulated surface called the fundamental wavelength surface is shown in Fig. 5 (a), which contains a single frequency component. Various filtration techniques for mean surface extraction are applied to this simulated surface. Figures 5 (b) and (c) show respectively the results of the areal Gaussian filter and the new areal spline filter, where *λ_xc_* = *λ_yc_* = 500 points and the boundary condition is selected to be periodic. For the calculation of the Gaussian filter, the data beyond the edges and the corners is assumed to be zero. The deviation between the two filtered results shown in Fig. 5 (d) indicates that the mean surfaces calculated by applying these two areal filters are almost identical, except for the ends of the surface. If we ignore the *λ_xc_*/2 or *λ_yc_*/2 surface distorted at the edges and the corners (which is caused by the standard Gaussian filter (Fig. 5 (b)), the actual maximum deviation is only 0.00073 µm and the largest relative error is less than 0.1 %. The result demonstrates both the isotropic characteristic and the ability for attenuating the end effects of the novel areal spline filter.

**Fig. 6:**
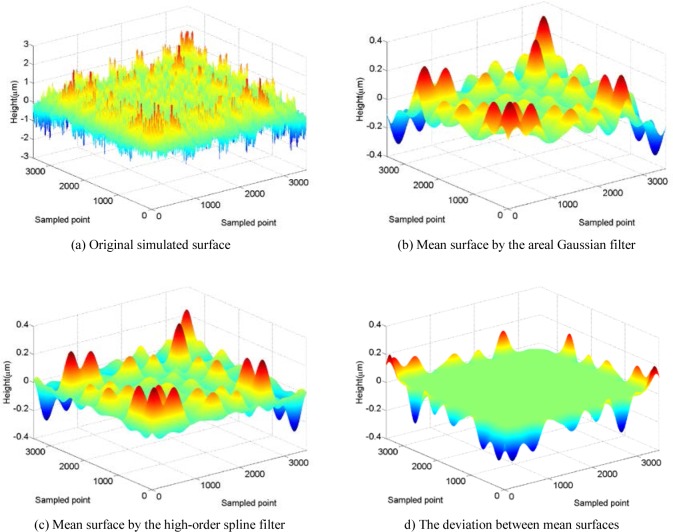
Simulated standard surface.

In order to further test the proposed spline filter, a more complicated simulated surface that consists of sinusoidal harmonic components is useful. Figure 6 (a) shows the simulated surface that is composed of 10 harmonic components in each orthogonal direction with frequencies ranging from 0.1/*λ_c_* to 25/*λ_c_.* Figures 6 (b) and (c) are the results determined by the areal Gaussian filter and the areal high-order spline filter, respectively, where the boundary condition is non-periodic. The difference between them shown in Fig. 6 (d) further illustrates that their filtering results are very close to each other except for those points at the boundary area. Not including those boundary area points, the maximum deviation between the two filtered results is 0.0046 μm, which is less than 0.1 % relative to the peak to peak value (2.3359 to −2.3359) of the simulated surface.

### 4.2 Practical Surface

**Fig. 7:**
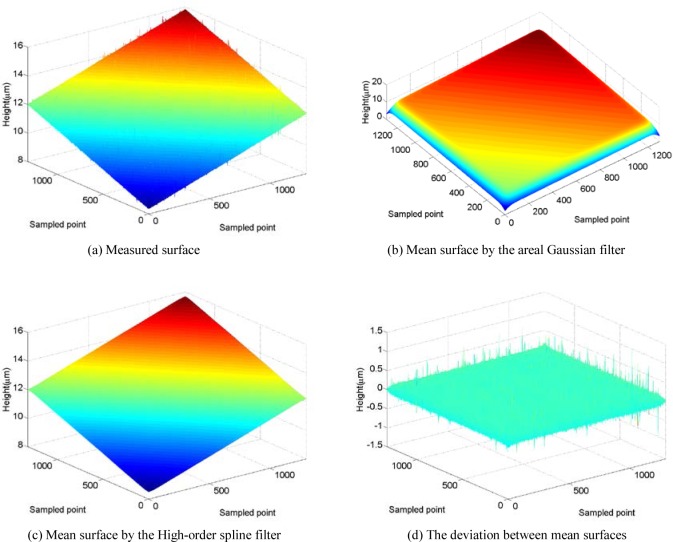
The original surface of standard calibrator.

Another example is given by applying the developed areal filter to an optical flat to validate its performance. The surface in Fig. 7 (a) is a standard calibrator surface with roughness parameter *R_a_* less than about 2.5 nm measured by a disk scanning confocal microscope. The measured area is 2.027 mm × 2.066 mm (1297 × 1322 sampling points). The cut-off wavelength *λ_xc_ = λ_yc_* = 312.56 μm, which is about 1/6 to 1/7 of the lateral measurement length in both directions. The filtered surface topography obtained with the Gaussian filter and the new spline filter are shown in Figs. 7 (b) and (c).

Although the deviation between the results from the two filters can be calculated as Fig. 7 (d) shows, it is difficult to evaluate the relative error, because the true amplitude value of the real surface is unknown. Fortunately, in Ref. [21], Song et al. introduce a practical method to make a useful topography comparison. A novel surface parameter called the relative topography difference, *D_s_*, is proposed for 2D and 3D surface topography measurement and comparison. “When *D_s_* = 0, the compared profiles or topographies must be exactly the same (point by point)”. The parameter *D_s_* and the cross-correlation function maximum *CCF_max_* are used to quantify and describe their difference clearly. Calculating the mean surfaces shown in Figs. 7 (b) and (c) with 1/2 cut-off wavelength trimmed on the edges, the results are obtained as *D_s_* = 0.000 745 % and *CCF_max_* = 0.999 993. Once again the results demonstrate that the areal high-order spline filter has an isotropic filtering characteristic like an areal Gaussian filter.

## 5. Conclusion

Despite the advantages of no end effect and fast calculation, the spline filter has not been widely used in the measurement of 3D surfaces because of the severely anisotropic characteristic of the areal spline filter.

In this paper, in order to overcome the problem of the anisotropic amplitude characteristic on successive implementation of the profile spline filter in the orthogonal directions, an approximation method is proposed. In this method, a high-order spline filter is constructed, and high-accuracy approximation to the filtering characteristic of the Gaussian filter can be achieved with increasing order of the spline filter. It is illustrated that the maximum deviation of the 3D transmission characteristic of the high-order spline filter from that of the Gaussian filter is only 1.1493 % when *n* = 4, and decreases to 0.5069 % when *n* = 5. Moreover, if a higher order is selected, the spline filter will have a transmission characteristic even closer to the Gaussian filter. A practical implementation of the areal spline filter is tested on two computer simulatedsurfaces and a smooth optical flat. As expected, the results reveal that the new areal spline filter not only possesses an isotropic transmission characteristic, but also inherits the ability to avoid end effects from the profile spline filtering algorithm.
